# Patents Associated with High-Cost Drugs in Australia

**DOI:** 10.1371/journal.pone.0060812

**Published:** 2013-04-05

**Authors:** Andrew F. Christie, Chris Dent, Peter McIntyre, Lachlan Wilson, David M. Studdert

**Affiliations:** 1 Melbourne Law School, University of Melbourne, Melbourne, Australia; 2 Health Innovations Research Institute, RMIT University, Melbourne, Australia; 3 UoM Commercial Ltd, University of Melbourne, Melbourne, Australia; 4 Melbourne School of Population and Global Health, University of Melbourne, Melbourne, Australia; Consejo Superior de Investigaciones Cientifics, Spain

## Abstract

Australia, like most countries, faces high and rapidly-rising drug costs. There are longstanding concerns about pharmaceutical companies inappropriately extending their monopoly position by “evergreening” blockbuster drugs, through misuse of the patent system. There is, however, very little empirical information about this behaviour. We fill the gap by analysing all of the patents associated with 15 of the costliest drugs in Australia over the last 20 years. Specifically, we search the patent register to identify all the granted patents that cover the active pharmaceutical ingredient of the high-cost drugs. Then, we classify the patents by type, and identify their owners. We find a mean of 49 patents associated with each drug. Three-quarters of these patents are owned by companies other than the drug's originator. Surprisingly, the majority of all patents are owned by companies that do not have a record of developing top-selling drugs. Our findings show that a multitude of players seek monopoly control over innovations to blockbuster drugs. Consequently, attempts to control drug costs by mitigating misuse of the patent system are likely to miss the mark if they focus only on the patenting activities of originators.

## Introduction

Like most countries, Australia faces high and rapidly-rising drug costs [1]. In the decade to 2010, the cost of prescription drugs covered by Australia's universal insurance scheme grew at 8% per annum to reach $8.4 billion [1]. The costs were remarkably concentrated: each year, the 10 drugs on which the government spent the most accounted for about a third of total drug expenditures, and the 25 costliest drugs accounted for about half of total drug expenditures [2]. The Australian situation is not unusual: a relatively small number of blockbuster drugs absorb a large proportion of pharmaceutical budgets in the United States and many other developed countries [3].

Most high-cost drugs enjoy patent protection. A key rationale for the patent system is that it creates incentives for socially-valuable research and innovation by granting inventors time-limited monopoly rights to make, use and sell their inventions, thereby providing them with the potential to recoup investments and reap profits. New drugs, particularly commercially successful ones, require large capital investments to develop, test and bring to market [4].

However, there are longstanding concerns about the misuse of patents by pharmaceutical companies to inappropriately extend their monopoly position [5]. Tactics such as “evergreening” and “patent thickets” have generated much speculation and debate [6], [7], [8], [9], [10], [11]. But aside from several widely-publicised examples of suspect patenting activity [12], there is virtually no empirical information identifying this behaviour, estimating its frequency, or revealing its nature. To the extent it does occur, misuse of drug patents may be both costly and inefficient for health systems.

This study analysed patenting activity around 15 of the costliest drugs in Australia over the last 20 years. Specifically, we determined the number, nature and ownership of these patents. The analysis included consideration of the patents granted to both the originator of the high-cost drugs under study and to other parties. Our goal was to contribute to the evidence base for understanding the potential misuse of patents in the pharmaceutical industry.

## Methods

### Identification of High-Cost Drugs

We used a publicly available source of information, the Australian Statistics on Medicines series [13], to identify a sample of the most costly drugs in Australia. Specifically, from among all drugs sold in Australia we calculated which 20 drugs accounted for the highest cumulative expenditures during the period 1990–2000. The expenditure data used to identify these “high-cost” drugs included both the subsidy paid by government and patients' out-of-pocket payments.

We wished to capture patents obtained after as well as before expiry of the original patent associated with each high-cost drug. We therefore dropped from further consideration any high-cost drug whose original patent had not expired by 31 December 2005 (n = 5). This left 15 drugs in the study sample. Table 1 describes the drugs and shows their cumulative costs over the period 1991–2008.

**Table 1:** Study sample of high-cost drugs.

Drug name	API Originator	Anatomical Therapeutic Chemical Classification	Cumulative cost 1991–2008 (millions) *
Simvastatin	Merck	HMG CoA reductase inhibitors – Lipid modifying agents, plain	$4,350
Omeprazole	AstraZeneca	Proton pump inhibitors – Drugs for peptic ulcer and gastro-oesophageal reflux disease	$2,917
Salbutamol sulfate	GSK	Selective beta-2-adrenoceptor agonists – Adrenergics, inhalants	$1,289
Ranitidine Hydrochloride	GSK	H2-receptor antagonists – Drugs for peptic ulcer and gastro-oesophageal reflux disease	$1,239
Enalapril Maleate	Merck	ACE inhibitors, plain	$1,115
Sertraline Hydrochloride	Pfizer	Selective serotonin reuptake inhibitors – Antidepressants	$1,090
Ipratropium Bromide	Boehringer Ingelheim	Anticholinergics – Other drugs for obstructive airway diseases, inhalants	$839
Felodipine	Astrazeneca	Dihydropyridine derivatives – Selective Calcium Channel Blockers with mainly vascular effects	$726
Budesonide	AstraZeneca	Glucocorticoids – Other drugs for obstructive airway diseases, inhalants	$647
Captopril	BMS	ACE inhibitors, plain	$614
Fluoxetine Hydrochloride	Eli Lilly	Selective serotonin reuptake inhibitors – Antidepressants	$583
Beclomethasone Dipropionate		Glucocorticoids – Other drugs for obstructive airway diseases, inhalants	$545
Glyceryl Trinitrate		Organic nitrates – Vasodilators used in cardiac diseases	$503
Famotidine	Merck	H2-receptor antagonists – Drugs for peptic ulcer and gastro-oesophageal reflux disease	$379
Cimetidine	GSK	H2-receptor antagonists – Drugs for peptic ulcer and gastro-oesophageal reflux disease	$190

* Totals are in 2008 Australian dollars. The cumulative costs should be interpreted in light of the fact that the study window caught drugs at different points in their commercial life cycle. Some drugs (e.g. Glyceryl Trinitrate) were being marketed before 1991; others (e.g. Felodipine) were not approved for marketing until later in the 1990s.

### Identification of Original Patent

We defined the “original patent” for each sampled drug as the patent in Australia on the drug's Active Pharmaceutical Ingredient (API). To identify the original patent, we searched the Merck Index [14] to obtain the patent number for the patent granted by the United States Patent and Trademark Office (USPTO) on the sampled drug's API. It is virtually certain that the patent on the API of all of the drugs in our sample would first or contemporaneously have been filed in the USPTO. Next, to identify the original patent in Australia, we used the PATSTAT [15] and INPADOC Patent Family and Legal Status [16] databases to match the USPTO patent number with the corresponding patent application filed in the Australian Patent Office (APO). For two drugs in our sample – Beclomethasone and Glyceryl Trinitrate – there was no original patent.

### Identification of Associated Patents

On various dates between May and August 2010, we searched the INPADOC Patent Family and Legal Status, Derwent World Patents Legal [17] and CAS SciFinder [18] databases for all patents, granted anywhere in the world at the date of the search, that contained a reference to the API of the drugs in our sample. To find the API we used its international non-proprietary name (INN) – a unique, globally recognised name assigned to the API by a specialist committee of the World Health Organization [19]. This set of searches produced a very large number of patent records.

We extracted the Australian records, eliminated duplicates, and identified the status of the patent application at the date we made the extraction (which was between May and August 2010). Many more drug patent applications are filed than are ultimately granted, often for commercial reasons (e.g. the patent application is filed prior to any clinical testing and regulatory approval of the drug, and is later abandoned when it becomes clear the prospective drug will not reach the market). We excluded all records for patent applications that had not proceeded to grant – i.e. that had not been examined and accepted by the APO – on the basis that only granted patents have legal effect. This process of elimination left a set of candidate patents for analysis.

To determine which of the candidate patents were actually associated with the sample drug named in their specification, we obtained the text of all published Australian patent specifications from AusPat Beta and AusPat [20], and from the State Library of Victoria, and then examined claim 1 in the patent specification. Claim 1 typically represents the broadest claim in a patent, and encompasses the fundamental concept of the invention. Thus, if claim 1 of a patent is not associated with a drug in our sample then it is almost certain that no other claim of that patent will be. We defined a patent as “associated” with a drug in our sample if claim 1 of that patent has an integer (i.e. an element of the claimed subject matter) that covers, or “reads onto”, the API of the drug. Determining the subject matter that an integer of a claim covers is an objective assessment routinely undertaken by patent lawyers and patent attorneys (and our research team had one of each).

A patent may be – and, in the case of our sample, sometimes was – associated with more than one drug (e.g. because claim 1 of the patent covered a combination of the APIs of two drugs in our sample). In that situation, we treated the patent as associated with each of those drugs. Because of the prevalence of such multiple associations in our sample, the total number of associated patents is greater than the total number of unique patents identified by our searching.

### Classification of Patents

We evaluated claim 1 of each patent associated with each drug in our sample to identify its nature. In doing so we observed claims on seven types of inventions: (1) the **API of the drug** (i.e. the drug's chemical compound); (2) an **intermediate or a different form of the API** (e.g. an isomer, or a salt or crystalline form, of the drug's chemical compound); (3) a **combination of the API**, or an intermediate or a different form of it, **with another drug** (e.g. the drug's chemical compound combined with the chemical compound of another drug); (4) a **delivery mechanism or a formulation for the API**, or an intermediate or a different form of it (e.g. a trans-dermal patch containing, or a slow-release formulation of, the drug's chemical compound; (5) a **process for making or formulating the API**, or an intermediate or a different form of it (e.g. a method of preparing or purifying the drug's chemical compound); (6) a **method of treatment using the API**, or an intermediate or a different form of it, for an indication in an Anatomical Therapeutic Chemical [21] (ATC) class the **same as the ATC** class of the indication for which the relevant sample drug was listed for government subsidy (e.g. a method of treating asthma using a drug that was subsidized for treatment of obstructive airway disease); and (7) a **method of treatment using the API**, or an intermediate or a different form of it, for an indication in an ATC class **different from the ATC** class of the indication for which the relevant sample drug was listed for government subsidy (e.g. a method of treating obesity using a drug that was subsidized for treatment of depression). A small number of patents were for inventions that did not fall into any of these seven categories (e.g. use of the API for a veterinary purpose), and were classified as “other”.

### Identification of Unique Patentees

The names of patent owners (“patentees”) were available in the databases used to identify the patents. After assembling a list of patentee names and eliminating duplicates, we checked whether each patentee on the list was linked to the corporate identity of another patentee through, for example, changes of company name, mergers and acquisitions, and holding company and subsidiary arrangements. Information to enable these checks came from the Mint Global [22] and Mergent [23] databases. When different patentee names proved to be of the same or a closely-linked entity, we collapsed them together under a common patentee name for classification purposes.

### Classification of Patentees

We classified the original patent, and any other patents held by the patentee of the original patent, as owned by the “API originator”. Two of the drugs in our sample had no original patent in Australia.

Patentees other than the API originator were classified into one of two groups. The first group consisted of patentees who held a patent on *another* high-cost drug (but not necessarily one from our sample). In this context, we defined a high-cost drug as any one of the 50 drugs associated with the largest cumulative expenditures in Australia over the period 1990–2000. We refer to a patentee in this group as an “other originator”; we refer to all the remaining patentees as a “non-originator”. The goal of separating patentees in this way was to allow for investigation of any differences between, on the one hand, a non-API originator who engages in research and development activities in relation to new drugs and, on the other hand, a non-API originator whose focus is on other activities, such as manufacture of generic drugs and upstream research.

In addition, because non-originator patentees were a group of particular interest, we sub-classified them into types of entities using information obtained from searches of the Australian Securities and Investment Commission and Bloomberg databases, Wikipedia, and the Internet. We found the non-originators to be of two types: pharmaceutical companies, and non-pharmaceutical organizations. If a pharmaceutical company either publicly identified itself as a generic manufacturer or was listed as a member of a generic pharmaceutical trade association, we classified it as “generic pharmaceutical company”; otherwise, we classified it as “other pharmaceutical company”. For the non-pharmaceutical organizations, we used four mutually-exclusive categories: “university”, “government agency”, “hospital”, “other”.

## Results

### Study Sample

**Figure 1:**
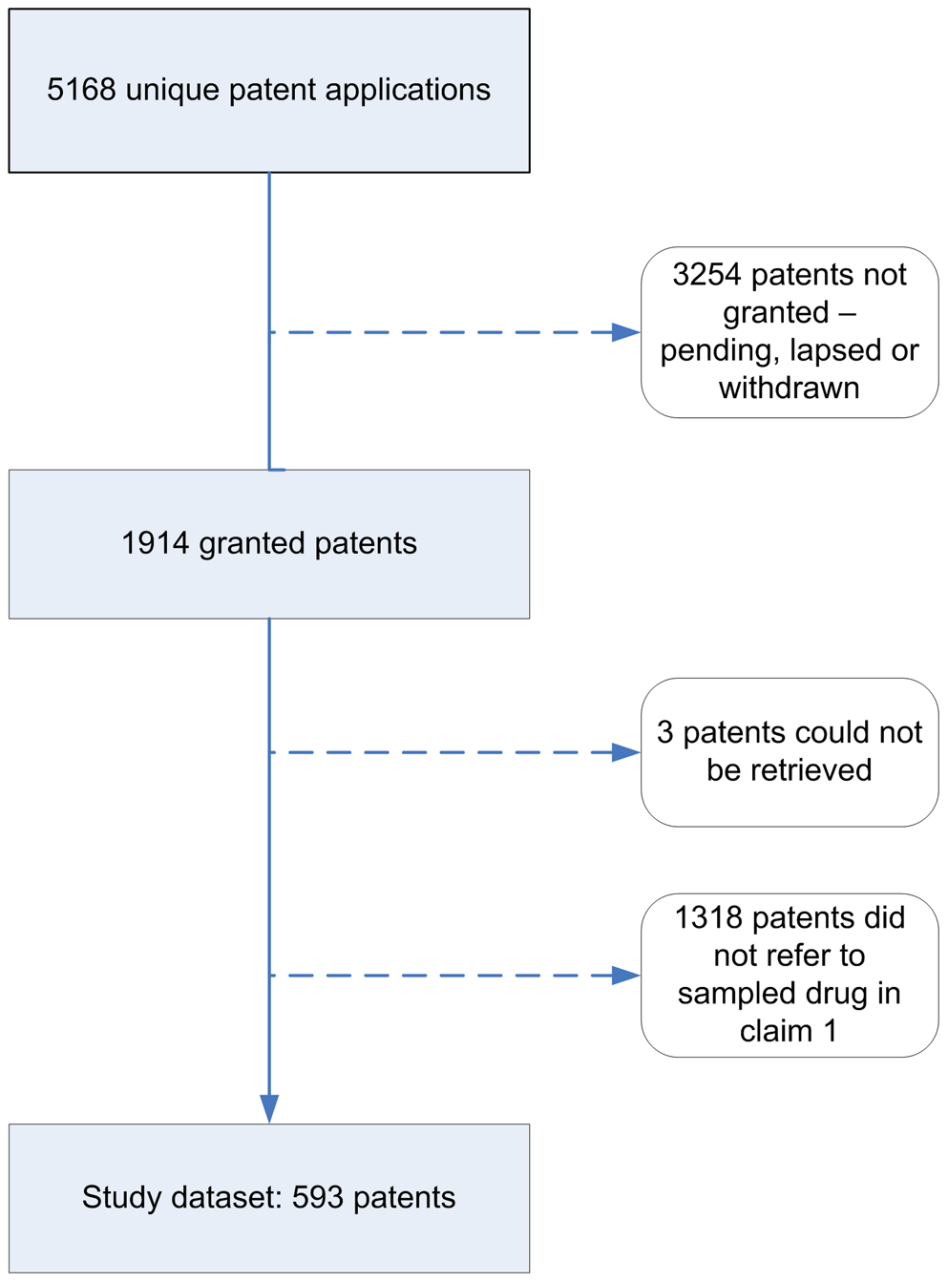
Identification of patents associated with high-cost drugs in the study sample. This flow chart shows the derivation of the patents associated with the drugs in our sample. The patents were selected by first identifying all unique patent applications that mentioned one of the high-cost drugs in our sample. There were found to be 5168 such applications in Australia, of which 3254 had not proceeded to grant – either because the application was pending, had lapsed or had been withdrawn. Of the remaining 1914, we could retrieve the specifications of all but three. Of the 1911 patent specifications analysed, 1318 had a claim 1 that did not read onto the API of a drug from the sample – leaving 593 unique patents associated with the drugs in our sample.

A total of 5168 unique patent applications mentioned the high-cost drugs in our sample, and 1914 (37%) of these applications were granted in Australia (Figure 1). We could locate the patent specification for all but three of the granted patents. Our review of those patent specifications showed that 593 of the granted patents were associated with one of the drugs in the sample. For the rest (n = 1318), claim 1 did not cover the API of any of the drugs.

### Patents by Drug

**Figure 2:**
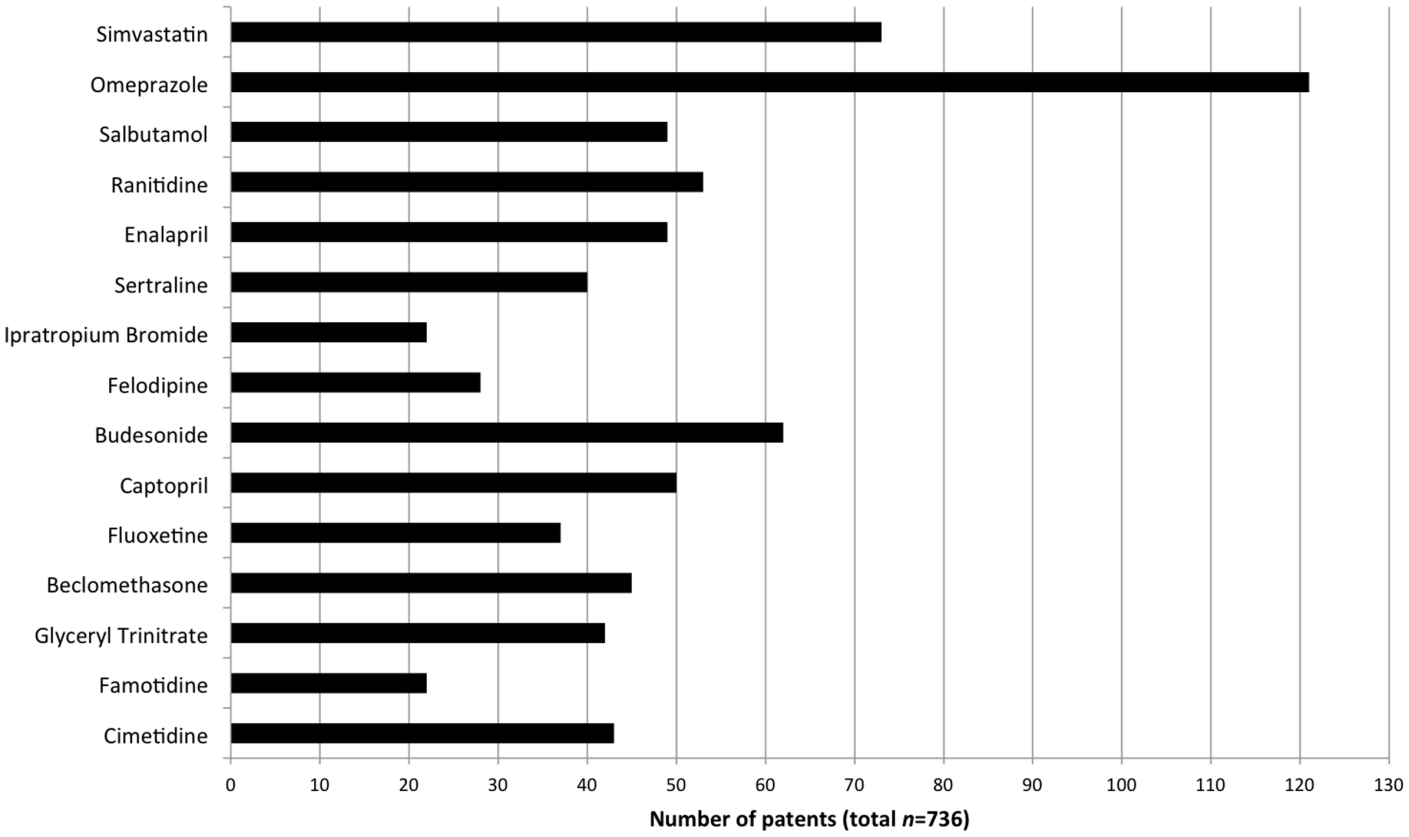
Patent counts by high-cost drug. This bar chart shows the count of patents associated with each high-cost drug in our sample. The drugs are arranged in descending order of total cumulative cost in the period 1991–2008.

The 593 unique patents had a total of 736 associations with drugs in our sample, producing a mean of 49 associated patents per drug (median 45). The number of associated patents per drug varied widely (standard deviation 24), ranging from 22 patents associated with Ipratropium and Famotidine to 121 patents associated with Omeprazole (Figure 2).

### Patents by Type of Patent and Patentee

**Figure 3:**
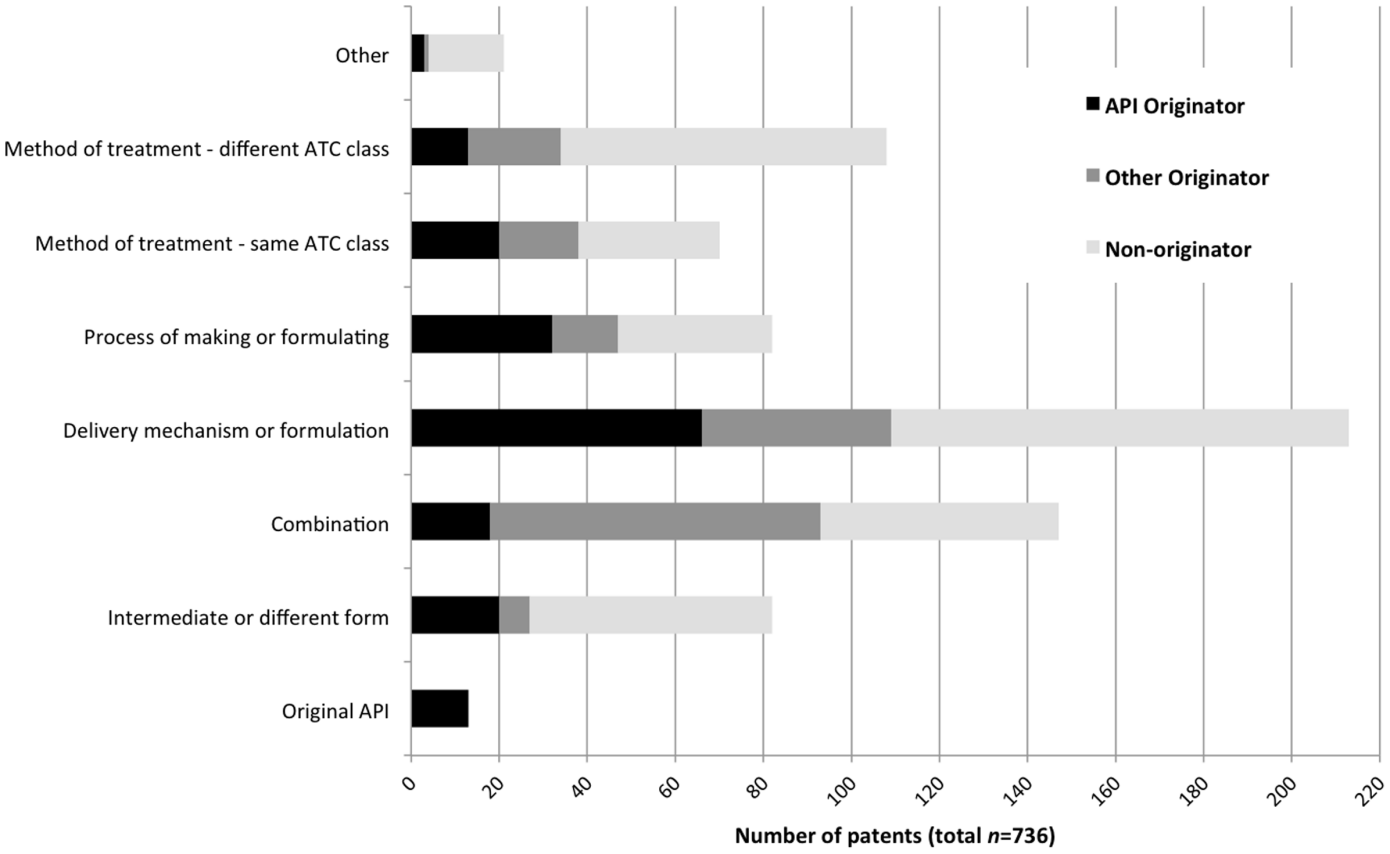
Patent counts by type of patent and patentee. This bar chart shows the count of associated patents in each category of patent, split by type of patent owner. For each bar, the black part counts patents owned by the ‘API originator’, the dark gray part counts patents owned by an ‘other originator’, and the light gray part counts patents owned by a ‘non-originator’.

Two types of patents accounted for one half (360/736) of all associated patents: 29% (213/736) were for a delivery mechanism or a formulation for the API, and 20% (147/736) were for a combination including the API (Figure 3). An additional 15% (108/736) of all associated patents were for a new method of treatment (different ATC class) using the API.

Patentees other than the API originator owned three-quarters (551/736) of all associated patents, and half of these patentees (367/736) were non-originators (i.e. entities that had not held patents on a top-50 high-cost drug in Australia from 1990–2000).

API originators were particularly active in patenting two types of inventions, which together accounted for just over one half (98/185) of all patents they owned: a delivery mechanism or a formulation for the API (36%); and a process for making or formulating the API (17%). API originators were not, however, the dominant patentee for either of these types of inventions: they owned only 31% and 39%, respectively, of all associated patents in these categories.

The patenting activities of other originators focused on combinations with the API; 41% of their associated patents were of this type. Other originators were the dominant patentee for this type of invention, owning 52% of all combination patents.

Non-originators were active in patenting inventions across the board. Excluding the original API (which was, by definition, held by the API originator), non-originators held more associated patents than either of the other patentees in every category except combinations. In addition, non-originators held more associated patents than API originators and other originators *combined* in two categories: a method of treatment (different ATC class) using the API (held 69% of all patents), and an intermediate or a different form of the API (67%).

### Non-originator Patentees

The 371 non-originator patents were held by 161 separate patentees, the vast majority (126/161) were pharmaceutical companies. Of the total number of non-originator patentees, we could identify 12% as generic pharmaceutical companies and 66% as other pharmaceutical companies. The remainder of the non-originator patentees were non-pharmaceutical organizations: universities (11%), government agencies (1%), hospitals (1%), and other entities (8%).

## Discussion

This study found a large number of patents encircled high-cost drugs in Australia. The majority of these patents relate to medicines that contain the API of the drug – either patents for a combination of the API with other pharmaceutical compounds, or patents for a delivery mechanism or a formulation for the API. Patents for a method of treatment (both same and different ATC class) using the API were also prevalent.

### Prevalence of Patents

The “one drug, one patent” perception is popular [24], but inaccurate. Only a few studies have actually sought to count the number of patents associated with pharmaceuticals, and these studies have produced widely varying results. Ouellette found an average of 3.5 patents per drug [24], and Hemphill and Sampat found an average of 2.7 patents per drug [6], based on a count of patents listed in the *Orange Book* under the U.S Hatch-Waxman linkage regime [25]. The European Commission's Pharmaceutical Sector Inquiry, using data reported to it by drug originators, found an average of 99 actual or potential patents per drug in its sample [26]; however, this figure included applications that had not (and might not) proceed to grant, and counted patents in multiple jurisdictions (i.e. in the 27 Member countries of the European Union). An analysis by Bouchard et. al. of a cohort of drugs to which the Canadian equivalent of the Hatch-Waxman linkage regime applied found an average of 40 patents granted per drug [27], while Amin and Kesselheim identified 82 granted US patents covering a sample of two drugs – an average of 41 patents per drug [28]. The patent count methods used in those two studies most closely resembles our method, and their findings regarding overall patent frequency by drug are also close to our findings.

However, the focus of all these previous studies has been confined to the patenting activities of the *originators* of the drugs of interest. Our findings indicate how large a piece of the picture of patenting behaviour this approach is likely to miss: among 736 patents connected to high-cost drugs in Australia, 3 in every 4 were by owned by entities other than the drug's originator. This highlights the importance of looking beyond patents disclosed in *Orange Book*-type listings. To identify the complete set of patents on major drugs, patent registers must be searched without restriction as to the owner of the patents.

### Types of Patent Owners

The prominence of patent owners other than the API originator in our findings may be surprising to some. We anticipated this result, although not its extent. What we did not anticipate, however, and which will surprise many, was the predominance of patent ownership among companies not engaged in the business of developing and marketing new top-selling drugs. It suggests that non-originator companies are investing substantial resources in follow-on innovations related to “blockbuster” drugs.

Through sub-analyses it was possible to attribute a profile to one-third of these non-originators, with the largest sub-group being generic pharmaceutical companies. For two-thirds of this group, however, it was not possible, within the scope of the study, to know more about them beyond that they were companies in the pharmaceutical industry. More research is needed to better understand the identity of this group, the nature of their patenting activities, and what part those activities play in their business models.

### Patenting Strategies

Examining patents associated with high-cost pharmaceuticals by type of invention sheds light on where the various industry participants have focused their investment. Our results show that the patents most commonly held by the API originator are for delivery mechanisms or formulations for the API, and for processes for making or formulating the API. The focus of API originators on these areas of innovation is probably not surprising, given that these areas are most closely connected with the original innovation, the API.

Patentees who were originators, but not the API originator, concentrated their patenting activity on medicines that combined the API with other compounds. A plausible explanation is that these companies are investing in follow-on innovation to combine their *own* drug compounds with the successful APIs of their competitors. It was beyond the scope of the current study to probe how often this explanation applied in the 75 combination patents owned by other originators, but it is interesting question for further research.

Non-originators patented heavily in three areas: delivery mechanisms or formulations for the API; methods of treatment (different ATC class); and intermediates or different forms of the API. The focus of non-originators on intermediate and alternative forms is logical: they are likely to be exploring these compounds in preparation for manufacture of the API once the original patent on it expires. However, the reasons behind non-originators' other areas of focus are less clear. The strategic value may be in providing leverage or bargaining power against the original patent holder should it seek to use its other associated patents to keep the non-originator out of the market after expiry of the original patent. The high levels of patenting activity we observed for both API originators and non-originators in the delivery mechanisms/formulations space, for example, points to this possibility. Qualitative research focused on non-originators' motivations would be needed to determine whether these or other motivations explain the patterns of patenting activity we observed.

### Limitations

The study has some limitations. First, we measured numbers of patents granted, but not the relative importance of those patents in conferring monopoly power over particular segments of the pharmaceutical market. Not all patents have equal commercial value. Classifying patents by type goes some way to filling in the picture, but it falls short of a precise grading of the commercial significance of the patents. That said, we know of no reliable method for doing such a grading.

Secondly, our study's findings come from an analysis of patents granted in Australia for high-cost drugs in Australia, and the ability to generalise to practices in other countries is unknown. Nevertheless, several factors support the generalisability of our findings. Multinational companies manufactured the drugs in our sample, and many appear consistently at or near the top of a “high-cost” drugs list in other developed countries. Thus, there are likely to be similar incentives elsewhere for obtaining patents on those drugs. Moreover, we are not aware of evidence that the pharmaceutical patenting standards in Australia are more lenient than those in other OECD countries. Nevertheless, the question of whether patenting practices around high-cost drugs in other countries conform to the patterns we observed warrants investigation.

## Conclusions

Commentary and policy discussions about inappropriate patenting behaviour, such as evergreening, have been centred on the originators of the drugs in question [5], [6], [8], [10], [11]. At one level, this is logical: as the owners of the original patent, they have the ability and incentives to extend their monopoly position. Implicit in this logic, however, is that mitigation of such behaviour by the originator will open the market to competitors and thereby drive efficiencies. The European Commission Inquiry put this view explicitly (para 461): “there have been no indications that patenting activities by generic companies, which are unlikely to hold a dominant position, would have negatively affected the possibility for generic or originator companies to enter the market” [26].

Our findings suggest that this account of patenting practices around high-cost drugs is too simplistic. It overlooks the substantial patenting activity undertaken by companies other than the originator of the high-cost drug, including generic manufacturers. Those other companies appear to be manoeuvring to stake out their own pockets of monopoly control for the period following expiration of the original patent on blockbuster drugs.

In the next few years, the original patents on a series of expensive drugs are due to expire. There is keen interest in how quickly greater access and efficiencies can be achieved [29]. Monitoring those important outcomes will demand attention to the behaviour of both the originators of those drugs and other companies scrambling to gain market control.
